# Preconditioning of Human Decidua Basalis Mesenchymal Stem/Stromal Cells with Glucose Increased Their Engraftment and Anti-diabetic Properties

**DOI:** 10.1007/s13770-020-00239-7

**Published:** 2020-02-19

**Authors:** Yasser Basmaeil, Manar Al Rashid, Tanvir Khatlani, Manal AlShabibi, Eman Bahattab, Meshan L. Abdullah, Fawaz Abumary, Bill Kalionis, Safia Massoudi, Mohammad AbuMaree

**Affiliations:** 1 Stem Cells and Regenerative Medicine Department, King Abdullah International Medical Research Center, King Abdulaziz Medical City, Ministry of National Guard Health Affairs, Mail Code 1515, P.O. Box 22490, Riyadh, 11426 Kingdom of Saudi Arabia; 2 National Center for Stem Cell Technology, Life Sciences and Environment Research Institute, King Abdulaziz City for Science and Technology, P.O Box 6086, Riyadh, 11442 Kingdom of Saudi Arabia; 3 Experimental Medicine, King Abdullah International Medical Research Center MNG-HA, Ali Al Arini, Ar Rimayah, Riyadh, 11481 Kingdom of Saudi Arabia; 4 Division of Obstetrics and Gynecology, Department of Clinical Science, Intervention and Technology, Karolinska Institutet, 14186 Stockholm, Sweden; 5 Department of Maternal-Fetal Medicine, Pregnancy Research Centre and University of Melbourne, Parkville, VIC 3010 Australia; 6 Department of Obstetrics and Gynaecology, Royal Women’s Hospital, 20 Flemington Rd, Parkville, VIC 3052 Australia; 7 Department of Forensic Biology, College of Forensic Sciences, Naif Arab University for Security Sciences, Khurais Rd, Ar Rimayah, Riyadh, 14812 Kingdom of Saudi Arabia; 8 College of Science and Health Professions, King Saud Bin Abdulaziz University for Health Sciences, King Abdulaziz Medical City, Ministry of National Guard Health Affairs, Mail Code 3124, P.O. Box 3660, Riyadh, 11481 Kingdom of Saudi Arabia

**Keywords:** Placental DBMSCs, Glucose, Cellular functions, Gene expression

## Abstract

**Background:**

Mesenchymal stem/stromal cells (MSCs) from the decidua basalis (DBMSCs) of the human placenta have important functions that make them potential candidates for cellular therapy. Previously, we showed that DBMSC functions do not change significantly in a high oxidative stress environment, which was induced by hydrogen peroxide (H_2_O_2_) and immune cells. Here, we studied the consequences of glucose, another oxidative stress inducer, on the phenotypic and functional changes in DBMSCs.

**Methods:**

DBMSCs were exposed to a high level of glucose, and its effect on DBMSC phenotypic and functional properties was determined. DBMSC expression of oxidative stress and immune molecules after exposure to glucose were also identified.

**Results:**

Conditioning of DBMSCs with glucose improved their adhesion and invasion. Glucose also increased DBMSC expression of genes with survival, proliferation, migration, invasion, anti-inflammatory, anti-chemoattractant and antimicrobial properties. In addition, DBMSC expression of B7H4, an inhibitor of T cell proliferation was also enhanced by glucose. Interestingly, glucose modulated DBMSC expression of genes involved in insulin secretion and prevention of diabetes.

**Conclusion:**

These data show the potentially beneficial effects of glucose on DBMSC functions. Preconditioning of DBMSCs with glucose may therefore be a rational strategy for increasing their therapeutic potential by enhancing their engraftment efficiency. In addition, glucose may program DBMSCs into insulin producing cells with ability to counteract inflammation and infection associated with diabetes. However, future *in vitro* and *in vivo* studies are essential to investigate the findings of this study further.

**Electronic supplementary material:**

The online version of this article (10.1007/s13770-020-00239-7) contains supplementary material, which is available to authorized users.

## Introduction

Mesenchymal stem /stromal cells (MSCs) are isolated from many human adult organs, including the placenta [1–3]. MSCs have multipotent differentiation potential [1–3] and possess immune-modulatory properties [4–7], which are essential for tissue restoration. Therefore, MSCs are considered attractive potential therapeutic agents to treat human diseases including diabetes, hypertension, and atherosclerosis [8–10]. In these inflammatory diseases, the environment is characterized by high levels of oxidative stress and inflammation. Consequently, for successful use of MSCs in these diseases, MSCs must maintain their normal functional activities to repair injured tissues in hostile microenvironments associated with oxidatively stress and inflammation. MSCs that cannot resist the toxic environment, are likely to have reduced therapeutic potential [11].

Recently, we reported the isolation and phenotypic characteristics of MSCs from the maternal decidua basalis tissue (DBMSCs) of human term placenta [1]. Human pregnancy is a condition where the maternal tissues and circulation are exposed to high levels of oxidative stress. DBMSCs in the maternal tissue of decidua basalis are a vascular microenvironment (i.e. niche), and are continuously exposed to high levels of oxidative stress products in the maternal circulation [12]. As a result, DBMSCs are conditioned to resist oxidative stress, as previously reported [13]. In a recent study, we showed that DBMSCs survive the harsh oxidative environment induced by high concentrations of H_2_O_2_, and that preconditioning of DBMSCs with H_2_O_2_ improved their functional activities [14]. In addition, that preconditioning of DBMSCs with H_2_O_2_ modulated their expression of genes with important cellular functions [14]. We also reported that DBMSCs protect endothelial cells functions from the damaging effects of both H_2_O_2_ and monocytes [15, 16]. Therefore, preconditioning of DBMSCs appears to be a rational approach for increase the efficiency of stem cell therapies associated with inflammatory diseases. In this study, we examined the functional responses of DBMSCs to another oxidative stress mediator; glucose. We exposed DBMSCs to high level of glucose and their phenotypic and functional properties were then assessed. We found that DBMSCs survived the harsh environment provided by high level of glucose, and that preconditioning of DBMSCs with glucose increased important functions, including adhesion and invasion. In addition, preconditioning of DBMSCs with glucose enhanced their expression of genes associated with various cellular functions including survival, proliferation, migration, invasion, immune modulation and microbial clearance. Glucose also increased DBMSC expression of B7H4, an immune protein with ability to inhibit T cell proliferation. Finally, DBMSC expression of genes involved in insulin secretion and prevention of diabetes was also modulated by glucose. These data indicate that glucose increases functions associated with engraftment of DBMSCs, and induced beneficial phenotypic changes in DBMSCs. We conclude that DBMSCs are potential candidate for the treatment of diabetes through a mechanism involving the reduction of inflammation and the secretion of insulin to lower glucose levels. However, more studies are essential to confirm these findings *in vitro* and *in vivo*.

## Materials and methods

### Ethical approval and the collection of tissues (human placentae and umbilical cord tissues)

This study was approved by the institutional review board (Reference # IRBC/246/13) at the King Abdulla International Medical Research Centre (KAIMRC). Human placentae and umbilical cord tissues were obtained from term, uncomplicated and healthy pregnancies (pregnant women have no medical problems, such as gestational diabetes or any other types of diabetes) after written consent from the donors. All clinical and experimental techniques in this study were conducted as per the guidelines and regulations of the KAIMRC. Placental tissues were immediately processed.

### Isolation and culture of DBMSCs and HUVEC (human umbilical vein endothelial cells)

DBMSCs were isolated from the decidua basalis which remains attached to the maternal side of the human term placenta after delivery, while HUVEC were isolated from umbilical cord veins as previously described by us [1, 16]. DBMSCs were cultured in a complete DBMSC culture medium [DMEM-F12 medium containing 10% MSCFBS (Mesenchymal Stem Cell certified fetal bovine serum, catalogue number 12-662-011, Life Technologies, Grand Island, NY, USA), and antibiotics (100 µg/mL streptomycin and 100 U/mL penicillin)], while HUVEC were cultured in a complete endothelial cell growth medium (Catalogue number PCS-100-041™, ATCC, Manassas, VA, USA). Cells (DBMSCs and HUVEC) were incubated at 37 °C in a humidified atmosphere containing 5% CO2 and 95% air (a cell culture incubator). DBMSCs (passage 3) and HUVEC (passages 3–5) of a total of 30 placentae and umbilical cords, respectively, were used in this study.

### DBMSC proliferation and adhesion in response to glucose

**Fig. 1:**
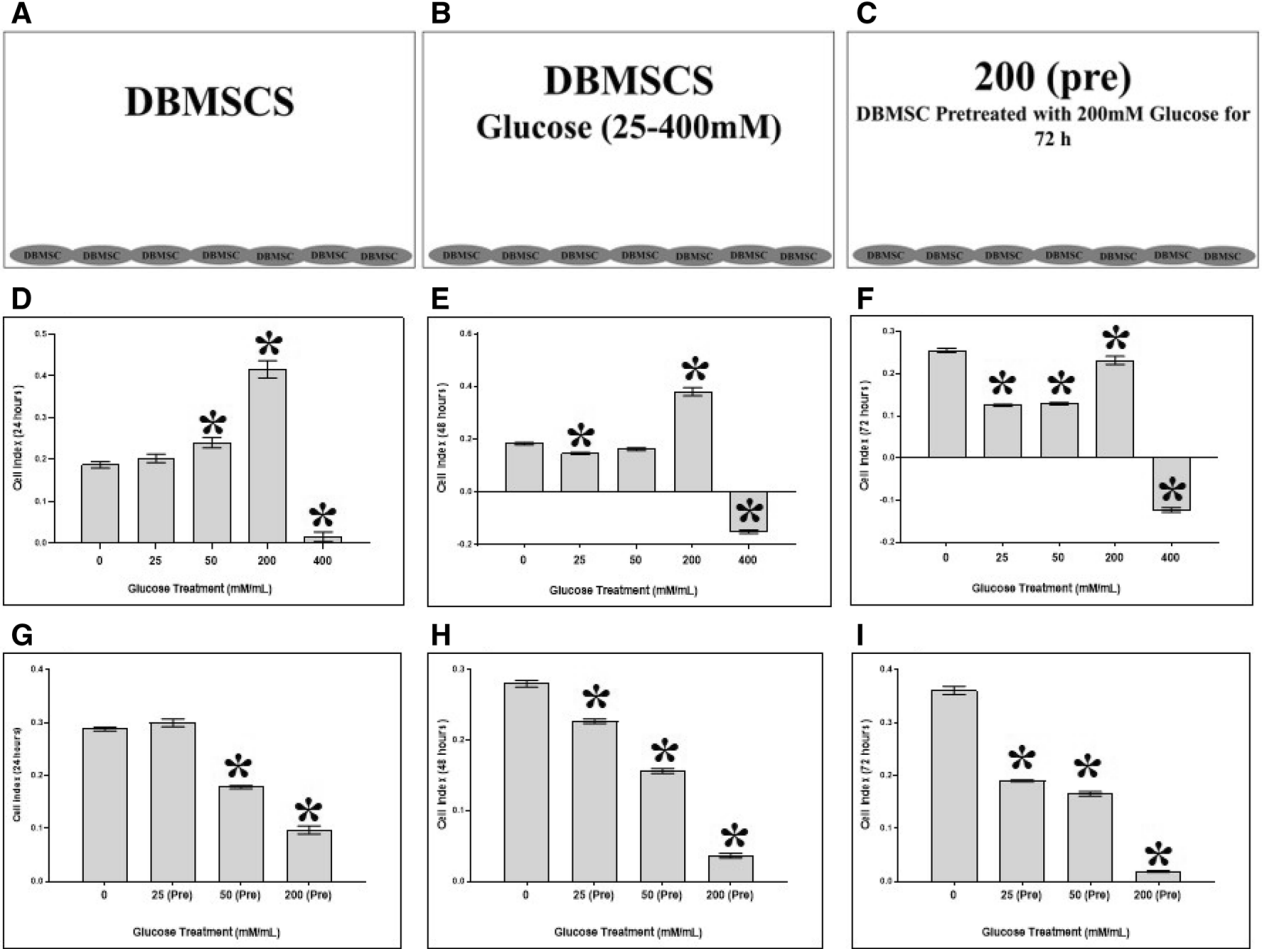
DBMSC proliferation groups. **A** Group 1 consisted of DBMSC cultured alone in a complete DBMSC culture medium. **B** Group 2 consisted of DBMSC cultured with different concentrations (25–400 mM) of glucose in a complete DBMSC culture medium. **C** Group 3 consisted of DBMSC pretreated with 200 mM glucose for 72 h [200 (pre)], harvested and then re-cultured alone in a complete DBMSC culture medium. **D** DBMSCs were seeded in a 16-well plate (E-Plate 16). The culture plates were then placed in the xCELLigence system at 37 °C in a cell culture incubator, and DBMSC cell index was then monitored. DBMSC proliferation in response to different glucose concentrations by the xCELLigence system. As compared to untreated DBMSCs, DBMSC proliferation was unchanged at 25 mM glucose (*p* > 0.05) but significantly increased at 50 and 200 mM glucose and then significantly reduced at 400 mM glucose, after 24 h in culture. **E** At 48 h in culture, and as compared to untreated DBMSCs, DBMSC proliferation was unchanged at 50 mM glucose (*p* > 0.05) but significantly increased at 200 mM glucose and then significantly reduced at 25 and 400 mM glucose. **F** At 72 h in culture, and as compared to untreated DBMSCs, DBMSC proliferation significantly increased at 200 mM glucose but was significantly reduced at 25, 50 and 400 mM glucose. **G**-**I** The reversibility of DBMSC proliferation in response to glucose. DBMSCs were initially cultured with 200 mM glucose for 72 h and their proliferation was then determined using the xCELLigence system. At 24–72 h in culture, and as compared to untreated DBMSCs and DBMSC-treated with 200 mM glucose [200 (I)], the proliferation of DBMSC pretreated with 200 mM glucose [200 (pre)] significantly reduced

DBMSC treatment groups consist of three groups as described in supplementry Table 1 and illustrated in Fig. 1A–C. The xCELLigence system (RTCA-DP version; Roche Diagnostics, Mannheim, Germany) was used to evaluate the adhesion and proliferation of HUVEC as we previously described [15–18]. Briefly, 2 × 104 DBMSCs were seeded in a complete DBMSC culture medium (as described above) containing different concentrations (25–400 mM) of glucose (Prince Care Pharma Pvt. Ltd, India) in 16-well culture plates (Catalogue number 05469813001, E-Plate 16, Roche Diagnostics). The culture plates were then placed in the xCELLigence system at 37 °C in a cell culture incubator and the DBMSC cell index was then monitored. The data for cell adhesion (at 2 h) and proliferation (24–72 h) was determined as previously described [15–18]. DBMSC viability was determined by Trypan blue exclusion. Each experiment was performed in triplicate and repeated with five independent DBMSC (passage 3) preparations.

### DBMSC migration in response to glucose

DBMSC migration was examined using the xCELLigence system and 16-well plates (Catalogue number 05665825001, CIM-16, Roche Diagnostics GmbH) as we described previously [15–18]. DBMSC treatment groups consist of three groups as described in supplementry Table 2 and illustrated in Fig. 3A–C. Briefly, 2 × 104 DBMSCs were seeded in the upper chamber, and the migration of cells was then monitored by the xCELLigence system [15–18]. The data were then expressed as a cell index value at 24 h. DBMSC migration with 30% FBS and without FBS served as positive and negative controls, respectively. Each experiment was performed and repeated as described above.

### DBMSC invasion under the effect of glucose

We evaluated the effect of glucose on the ability of DBMSCs to invade through a monolayer of endothelial cells using the xCELLigence system. Briefly, 2 × 104 HUVEC seeded in a complete endothelial cell growth medium in a 16-well culture E-Plate (as described above) until cells reached a growth plateau (20 h). Different treatments of DBMSCs (supplementary Table 3) were then added to the monolayer of endothelial cells. At 10 h, the data for the invasion recorded and expressed as a cell index (mean ± standard error). The rate of cell invasion was determined by calculating the normalized cell index at pausing time (20 h) of HUVEC growth.

### Gene expression by real-time polymerase chain reaction (RT-PCR)

DBMSC expression of 84 genes related to Human Oxidative Stress (Catalogue # PAHS-065ZD, Qiagen, Hilden, Germany) was identified using our previously published method [1, 14, 15, 18]. Total RNA was extracted from DBMSCs pretreated with 200 mM glucose for 72 h, and cDNA was then synthesized and used in a QuantiTect Primer Assay (Qiagen, Hilden, Germany). The real-time polymerase chain reaction (RT-PCR) was performed in triplicate on the CFX96 real-time PCR detection system (BIO-RAD, Hercules, CA, USA), and the data was then analysed as previously described [1, 14, 15, 18]. The ΔΔ^−2^ values were then calculated to express the results as fold changes. The relative expression of internal controls (house-keeping genes) were used as provided in the kit. Experiments were performed in triplicate and repeated three times using DBMSCs prepared from three independent placentae.

### Flow cytometry

DBMSCs (1 × 105) were stained with antibodies for ICAM-1, IL-12, and B7H4 for 30 min and then flow cytometry was performed as previously described [1]. Negative controls were cells stained with FITC or PE- labelled mouse IgG isotype antibody.

### Statistical analysis GraphPad Prism 5 was used to analyze data using non-parametric tests

(Mann–Witney U and Kruskal–Wallis). Data were deemed statistically significant if *p* < 0.05.

## Results

### Glucose effect on DBMSC proliferation

DBMSCs were isolated using our established published method [1, 14–16] to assess the effect of glucose on their proliferation using the xCELLigence system. At 24 h, and as compared to untreated DBMSCs, the proliferation of DBMSCs unchanged at 25 mM glucose (*p* > 0.05), significantly increased at 50 and 200 mM glucose (*p* < 0.05), and was significantly reduced at 400 mM glucose, *p* < 0.05 (Fig. 1D). At 48 h, and as compared to untreated DBMSCs, the proliferation of DBMSCs unchanged at 50 mM glucose (*p* > 0.05), significantly increased at 200 mM glucose (*p* < 0.05), and significantly reduced at 25 and 400 mM glucose, *p* < 0.05 (Fig. 1E). Finally, at 72 h, and as compared to untreated DBMSCs, the proliferation of DBMSCs, significantly increased at 200 mM glucose (*p* < 0.05) but was significantly reduced at 25, 50 and 400 mM glucose, *p* < 0.05 (Fig. 1F). The viability of DBMSC treated with glucose (25–200 mM) for 72 h was >90% as determine by Trypan Blue exclusion. At 96 h, treatment with glucose (25–200 mM) reduced the viability of DBMSCs (< 50%) while the treatment with 400 mM glucose reduced the viability of DBMSCs (< 50%) at all examined culture times (24–72 h) (supplementary Fig. 1). Based on the results obtained above, the exposure time of 72 h and glucose at concentration of glucose (200 mM) was selected to evaluate the effect of glucose on DBMSC functions.

### Reversibility of glucose effect on DBMSC proliferation

To evaluate the reversibility of the glucose effect on DBMSC proliferation, DBMSCs were initially cultured with 200 mM glucose for 72 h and their proliferation was then determined using the xCELLigence system. As compared to untreated DBMSCs and DBMSC-treated with 200 mM glucose during the proliferation experiment [200 (pre)], the proliferation of DBMSC pretreated with 200 mM glucose [200 (pre)] significantly reduced (*p* < 0.05) at all examined culture times (24–72 h), (Fig. 1G–I). These results show the effect of glucose on DBMSC proliferation is reversible.

### Glucose effect on DBMSC adhesion

**Fig. 2:**
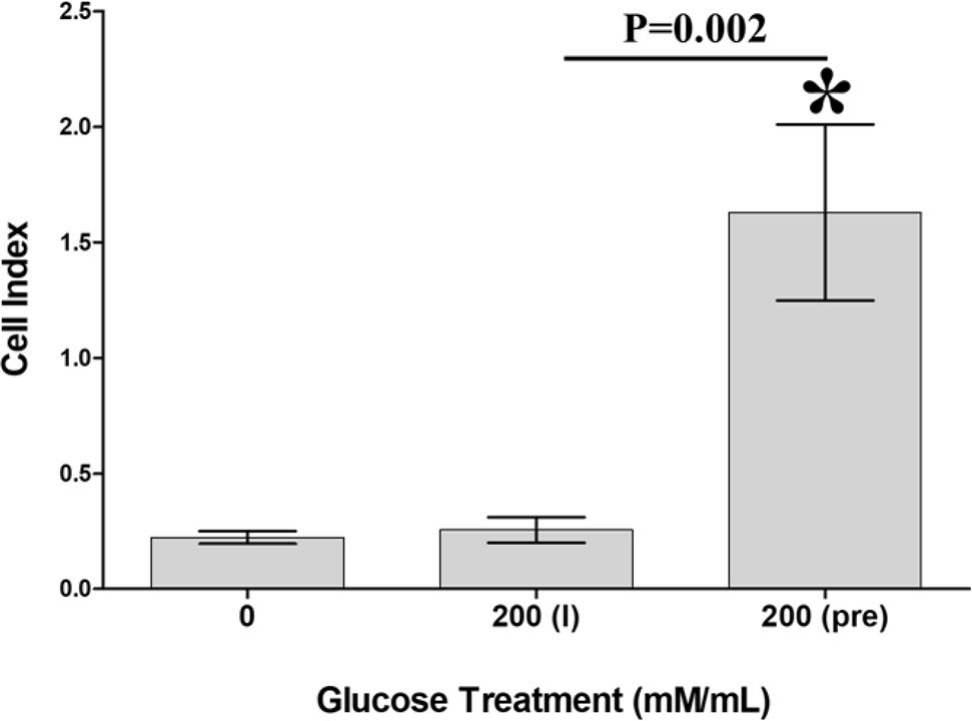
BMSCs were cultured with 200 mM glucose [200 (I)] and their adhesion was then determined using the xCELLigence system. As compared to untreated DBMSCs, the treatment with 200 mM glucose had no significant effect on DBMSC adhesion at 2 h in culture (*p* > 0.05) while the adhesion of DBMSC pretreated with 200 mM glucose for 72 h was significantly increased at 2 h as compared to untreated DBMSCs, and DBMSC cultured with 200 mM glucose [200 (I)]. Each experiment was performed in triplicate and repeated with five independent DBMSC (passage 3) preparations. *p < 0.05. Bars represent standard errors

To study the effects of glucose on the adhesion of DBMSCs, DBMSCs were cultured with 200 mM glucose and their adhesion was then determined using the xCELLigence system. At 2 h, and as compared to untreated DBMSCs, the adhesion of DBMSCs unchanged after treatment with 200 mM glucose, *p* > 0.05 (Fig. 2).

We also evaluated the reversibility of glucose effects on DBMSC adhesion. DBMSCs were initially cultured with 200 mM glucose for 72 h and their adhesion was then determined using the xCELLigence system. After 2 h, and as compared to untreated DBMSCs, and DBMSC-treated with 200 mM glucose during the experiment [200 (I)], the adhesion of DBMSCs pretreated with 200 mM glucose [200 (pre)] significantly increased, *p* < 0.05 (Fig. 2).

### Glucose effect on DBMSC migration

**Fig. 3:**
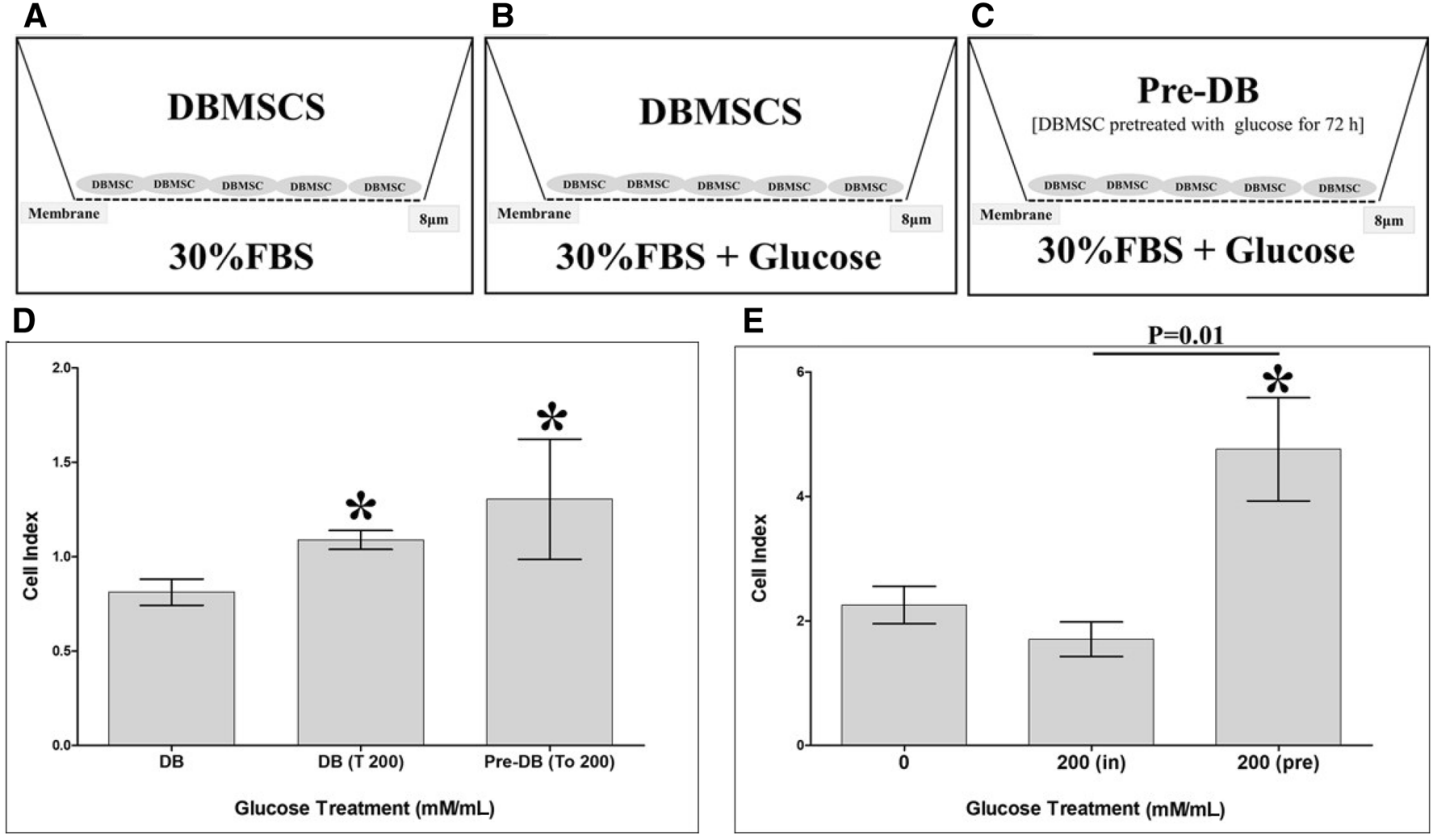
DBMSC migration groups. **A** Group 1 consisted of DBMSCs cultured alone in the upper chamber. **B** Group 2 consisted of DBMSCs cultured alone in the upper chamber while 200 mM glucose was added to the lower chamber. **C** Group 3 consisted of DBMSCs pre-treated with 200 mM glucose for 72 h (200(pre)) harvetsed and then re-cultured alone in the upper chamber while 200 mM glucose was added to the lower chamber. **D** DBMSCs were seeded in DBMSC serum free medium in the upper chamber of the CIM migration plate while DBMSC culture medium containing 30% FBS was added to the lower chambers. At 24 h, DBMSC [DB (T 200)] migration in response to 200 mM glucose significantly increased as compared to untreated DBMSCs (DB). The migration of DBMSCs pretreated with 200 mM glucose for 72 h (Pre-DB) in response to 200 mM glucose [Pre-DB (To 200)] significantly increased as compared to untreated DBMSCs (DB), but was unchanged as compared to DBMSC migrating in response to 200 mM glucose [DB (To 200)], *p* > 0.05. **E** The effect of glucose on DBMSC invasion through endothelial cells by the xCELLigence system. At 10 h, the pretreatment with 200 mM glucose for 72 h [200 (Pre)] significantly increased DBMSC invasion as compared to untreated DBMSCs and DBMSC cultured with 200 mM glucose while the addition of 200 mM glucose [200 (in)] during the invasion experiment had no significant effect on DBMSC invasion as compared to untreated DBMSCs. Each experiment was performed in triplicate and repeated with five independent DBMSC (passage 3) preparations. *p < 0.05. Bars represent standard errors

To further study the effect of glucose on DBMSC functions, the migration of DBMSCs was monitored using the xCELLigence system. At 24 h and as compared to untreated DBMSCs (DB), the migration of DBMSCs in response to 200 mM glucose [DB (To 200)] significantly increased, *p* < 0.05 (Fig. 3D).

We also evaluated the reversibility of glucose effects on DBMSC migration, DBMSCs were initially cultured with 200 mM glucose for 72 h and their migration was then determined using the xCELLigence system. The migration of DBMSC-pretreated with 200 mM glucose for 72 h in response to 200 mM glucose [Pre-DB (To 200)] significantly increased (*p* < 0.05) as compared to untreated DBMSCs (DB) but unchanged as compared to DBMSCs-migrated in response to 200 mM glucose [DB (To 200)], *p* > 0.05 (Fig. 3D). These results show the effect of glucose on DBMSC migration is irreversible.

### Glucose effect on DBMSC invasion

We also evaluated the effect of glucose on DBMSC invasion through endothelial cells using the xCELLigence Real-Time Cell Analyser. In the xCELLigence Rea-Time system, increased invasion is defined as an increase in the cell index due to the infiltration of HUVEC monolayer by DBMSCs and this therefore causing detachment of HUVEC while the increased of DBMSC adhesion is reflected by the increased of cell index defining the increase in cell invasion. At 10 h and as compared to untreated DBMSCs and DBMSCs cultured with 200 mM glucose, the pre-treatment of DBMSCs with 200 mM glucose for 72 h [200 (Pre)] significantly increased DBMSC invasion, *p* < 0.05 while the addition of 200 mM glucose [200 (in)] during the course of invasion experiment had no significant effect on DBMSC invasion, *p* > 0.05 (Fig. 3E).

### Glucose increases DBMSC expression of adhesion and anti-inflammatory markers

**Fig. 4:**
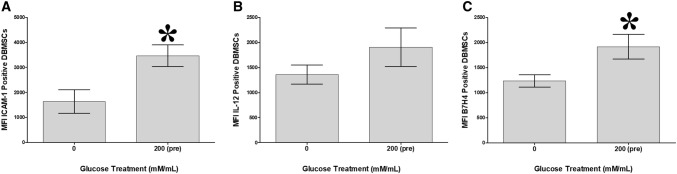
Flow cytometric analysis of DBMSC expression of immune markers. **A**-**C** The treatment with 200 mM glucose significantly increased the DBMSCs [200 (pre)] expression of ICAM-1, had no significant effect on IL-12 expression, *p* > 0.05, and significantly increased the expression of B7H4, as compared untreated DBMSCs. Each experiment was performed in triplicate and repeated with five independent DBMSC (passage 3) preparations. *p < 0.05. Bars represent standard errors

To evaluate the modulatory effects of glucose on DBMSC functions, a variety of immune proteins important in DBMSC functions were studied by flow cytometry and expression recorded as median fluorescence intensity. As compared to untreated DBMSCs, after incubation with 200 mM glucose for 72 h [200 (pre)], DBMSC expression of ICAM-1 and B7H4, significantly increased *p* < 0.05 (Fig. 4), while the expression of IL-12 by DBMSCs did not significantly change, *p* > 0.05 (Fig. 4B).

### Glucose modulated the expression of genes important in DBMSC functions

The expression of oxidative stress-associated genes by DBMSCs was studied after culturing DBMSCs with 200 mM glucose for 72 h, and then analysed and assessed using the RT-PCR. Results show that glucose modulated DBMSC expression of variety of genes associated with many cellular functions as compared to untreated DBMSC as shown in Tables 1, 2, 3, 4 and 5.

**Table 1:** Glucose effects on DBMSC expression of oxidative genes with survival, anti-apoptotic, proliferation, and migration properties. DBMSCs were untreated (DBMSC) or treated with 200 mM glucose (TDBMSC) for 72 h

#	Gene symbol	Gene full name	DBMSC Mean ΔΔ^−2^ values	TDBMSC Mean ΔΔ^−2^ values	Fold change (TDBMSC vs. DBMSC) *p* < 0.05		Biological properties
1	GPX2	Glutathione peroxidase 2	1	5	1 fold	↑	1. Survival property 2. Anti-apoptotic property 3. Migration property 4. Invasion property
2	GPX3	Glutathione peroxidase 3	1	16	16 fold	↑	
3	GPX4	Glutathione peroxidase 4	1	60	60 fold	↑	
4	GPX7	Glutathione peroxidase 7	1	4	4 fold	↑	
5	PRDX1	Peroxiredoxin 1	1	1.5	1.5 fold	↑	1. Survival property 2. Migration property 3. Invasion property
6	PRDX4	Peroxiredoxin 4	1	2.52	2.52 fold	↑	
7	PRDX5	Peroxiredoxin 5	1	2.63	2.63 fold	↑	
8	PRDX6	Peroxiredoxin 6	1	40	40 fold	↑	
9	HMOX1	Heme oxygenase- 1	1	3.18	3.18 fold	↑	1. Survival property 2. Migration property
10	ALB	Albumin	1	3	3 fold	↑	Survival property
11	OXR1	Oxidation resistance 1	1	12	12 fold	↑	
12	KRT1	Keratin 1	1	2618	2618 fold	↑	
13	NQO1	NAD(P)H dehydrogenase, quinone 1	1	21	21 fold	↑	
14	MB	Myoglobin	1	707	707 fold	↑	
15	STK25	Serine/threonine kinase 25	1	77	77 fold	↑	1. Anti-apoptotic property 2. Migration property
16	ALOX12	Arachidonate 12-lipoxygenase	1	519	519 fold	↑	1. Proliferation property 2. Migration property 3. Invasion property
17	TFII-I (GTF2I)	General transcription factor 2I	1	60	60 fold	↑	Proliferation property
18	FOXM1	Forkhead box M1	1	34	34 fold	↑	
19	BNIP3	BCL2/adenovirus E1B 19 kDa interacting protein 3	1	49	49 fold	↑	Migration property

**Table 2:** Glucose effects on DBMSC expression of oxidative genes with pro-oxidant and antioxidant properties. DBMSCs were untreated (DBMSC) or treated with 200 mM glucose (TDBMSC) for 72 h

#	Gene symbol	Gene full name	DBMSC Mean ΔΔ^−2^ values	TDBMSC Mean ΔΔ^−2^ values	Fold change (TDBMSC vs. DBMSC) *p* < 0.05		Biological properties
1	NOX2 (CYBB)	Cytochrome b-245, β polypeptide	1	11.62	11.62 fold	↑	Pro-oxidant property
2	NOX4	NADPH oxidase 4	1	9.89	> 9 fold	↑	
3	NOX5	NADPH oxidase 5	1	14	14 fold	↑	
4	DUOX1	Dual oxidase 1	1	2	2 fold	↑	
5	DUOX2	Dual oxidase 2	1	52	52 fold	↑	
6	NCF1	Neutrophil cytosolic factor 1	1	6.59	6.59 fold	↑	
7	NCF2	Neutrophil cytosolic factor 2	1	50	50 fold	↑	
8	ALOX12	Arachidonate 12-lipoxygenase	1	519	519 fold	↑	
9	AOX1	Aldehyde oxidase 1	1	34	34 fold	↑	
10	GPX1	Glutathione peroxidase 1	1	0.10	10 fold	↓	Anti-oxidant property
11	GPX5	Glutathione peroxidase 5	1	13	13 fold	↓	
12	GPX6	Glutathione peroxidase 6	1	0.02	50 fold	↓	
13	PRDX2	Peroxiredoxin 2	1	0.02	50 fold	↓	
14	PRDX3	Peroxiredoxin 3	1	0.39	> 2.50 fold	↓	
15	CAT	Catalase	1	0.56	> 1.70 fold	↓	
16	SOD1	Superoxide dismutase 1	1	0.016	> 62 fold	↓	
17	SOD2	Superoxide dismutase 2	1	0.052	> 19 fold	↓	
18	TTN	Titin	1	0.20	5 fold	↓

**Table 3:** Glucose increased DBMSC expression of genes with antioxidant, anti-inflammatory, anti-chemoattractant, and antimicrobial properties. DBMSCs were untreated (DBMSC) or treated with 200 mM glucose (TDBMSC) for 72 h

#	Gene symbol	Gene full name	DBMSC Mean ΔΔ^−2^ values	TDBMSC Mean ΔΔ^−2^ values	Fold change (TDBMSC vs. DBMSC) *p* < 0.05		Biological properties
1	GPX2	Glutathione peroxidase 2	1	5	1 fold	↑	Anti-oxidant property 2. Anti-inflammatory property
2	GPX3	Glutathione peroxidase 3	1	16	16 fold	↑	
3	GPX4	Glutathione peroxidase 4	1	60	60 fold	↑	
4	GPX7	Glutathione peroxidase 7	1	4	4 fold	↑	
5	UCP2	Uncoupling protein 2	1	7	7 fold	↑	
6	SEPP1	Selenoprotein P, plasma, 1	1	10	10 fold	↑	
7	PRDX1	Peroxiredoxin 1	1	1.5	1.5 fold	↑	Anti-oxidant property
8	PRDX4	Peroxiredoxin 4	1	2.52	2.52 fold	↑	
9	PRDX5	Peroxiredoxin 5	1	2.63	2.63 fold	↑	
10	PRDX6	Peroxiredoxin 6	1	40	40 fold	↑	
11	TPO	Thyroid peroxidase	1	2.48	2.48 fold	↑	
12	TFII-I (GTF2I)	General transcription factor 2I	1	60	60 fold	↑	
13	BNIP3	BCL2/adenovirus E1B 19 kDa interacting protein 3	1	49	49 fold	↑	
14	PNKP	Polynucleotide kinase 3’-phosphatase	1	2.75	2.75 fold	↑	
15	SOD3	Superoxide dismutase 3	1	4.45	4.45 fold	↑	Anti-oxidant property Anti-inflammatory property 3. Anti-chemoattractant property
16	HMOX1	Heme oxygenase- 1	1	3.18	3.18 fold	↑	Anti-inflammatory property
17	MT3	Metallothionein 3	1	124	124 fold	↑	
18	GSR	Glutathione reductase	1	2.55	2.55 fold	↑	
19	DUSP1	Dual specificity phosphatase 1	1	2	2 fold	↑	
20	KRT1	Keratin 1	1	2618	2618 fold	↑	
21	NQO1	NAD(P)H dehydrogenase, quinone 1	1	21	21 fold	↑	
22	SIRT2	Sirtuin 2	1	2.99	2.99 fold	↑	
23	SFTPD	Surfactant protein D	1	114	114 fold	↑	Anti-inflammatory property 2. Antimicrobial property
24	MBL2	Mannose-binding lectin (protein C) 2	1	6.34	6.34 fold	↑

**Table 4:** Glucose effects on DBMSC expression of oxidative genes. DBMSCs were untreated (DBMSC) or treated with 200 mM glucose (TDBMSC) for 72 h

#	Gene symbol	Gene full name	DBMSC Mean ΔΔ^−2^ values	TDBMSC Mean ΔΔ^−2^ values	Fold change (TDBMSC vs. DBMSC) *p* < 0.05		Biological properties
1	CYGB	Cytoglobin	1	0.56	> 1.70 fold	↓	Cytoprotective property
2	TXNRD2	Thioredoxin reductase 2	1	18	18 fold	↑	Anti-growth property
3	NOS2	Nitric oxide synthase 2	1	15.81	15.81 fold	↑	Inflammatory property
4	MGST3	Microsomal glutathione S-transferase 3	1	0.34	> 2.9 fold	↓	
5	COX1	Cyclooxygenase 1	1	2	2 fold	↑	
6	COX2	Cyclooxygenase 2	1	0.0077	> 129 fold	↓	
7	NOX4	NADPH oxidase 4	1	9.89	> 9 fold	↑	
8	NOX5	NADPH oxidase 5	1	14	14 fold	↑	
9	GSTZ1	Glutathione transferase zeta 1	1	0.0002	5000 fold	↓	Anti-inflammatory property
10	GSTP1	Glutathione S-transferase pi 1	1	0.06	> 16	↓	Anti-apoptotic Property 2. Anti-inflammatory property
11	UCP2	Uncoupling protein 2	1	7	7 fold	↑	Inhibits Insulin Secretion
12	PXDN	Peroxidasin homolog	1	0.24	> 4 fold	↓	Triggers Diabetes
13	BNIP3	BCL2/adenovirus E1B 19 kDa interacting protein 3	1	49	49 fold	↑	Pro-apoptotic property
14	PRNP	Prion protein	1	0.05	20 fold	↓	Anti-inflammatory property
15	HSP70-1A	Heat shock 70 kDa protein 1A	1	0.05	20 fold	↓	
16	PDLIM1	PDZ and LIM domain 1	1	0.01	100 fold	↓	
17	TTN	Thioredoxin	1	0.10	10 fold	↓	Chemoattractant property
18	SOD1	Superoxide dismutase 1	1	0.016	> 62 fold	↓	Antioxidant property Anti-inflammatory property 3. Anti-chemoattractant property
19	SOD2	Superoxide dismutase 2	1	0.052	> 19 fold	↓	
20	LPO	Lactoperoxidase	1	0.029	> 34 fold	↓	Antimicrobial property 2. Oxidative property

**Table 5:** Glucose effects on DBMSC expression of oxidative genes. DBMSCs were untreated (DBMSC) or treated with 200 mM glucose (TDBMSC) for 72 h

#	Gene symbol	Gene full name	DBMSC Mean ΔΔ^−2^ values	TDBMSC Mean ΔΔ^−2^ values	Fold change (TDBMSC vs. DBMSC)
1	EPX	Eosinophil peroxidase	1	1.09	Fold Change is not statistically significant, *p* > 0.05
2	APOE	Apolipoprotein E	1	1.26	
3	TXNRD1	Thioredoxin reductase 1	1	0.74	
4	EPHX2	Epoxide hydrolase 2, cytoplasmic	1	0.81	
5	APOE	Apolipoprotein E	1	1.26	
6	GCLM	Glutamate-cysteine ligase, modifier subunit	1	0.88	
7	GPX1	Glutathione peroxidase 1	1	0.10	
8	FTH1	Ferritin, heavy polypeptide 1	1	0.62	
9	EPX	Eosinophil peroxidase	1	1.09	
10	MSRA	Methionine sulfoxide reductase A	1	0.71	
11	OXSR1	Oxidative-stress responsive 1	1	0.88	
12	RNF7	Ring finger protein 7	1	0.61	
13	SCARA3	Scavenger receptor class A, member 3	1	0.79	
14	SQSTM1	Sequestosome 1	1	0.65	
15	NUDT1	Nudix (nucleoside diphosphate linked moiety X)-type motif 1	1	0.37	
16	SRXN1	Sulfiredoxin	1	1.72	
17	PREX1	Phosphatidylinositol-3,4,5-trisphosphate-dependent Rac exchange factor 1	1	1.59	
18	CCS	Copper chaperone for superoxide dismutase	1	0.65	
19	DHCR24	24-dehydrocholesterol reductase	1	1.40	
20	GCLC	Glutamate-cysteine ligase, catalytic subunit	1	1.90	
21	GSS	Glutathione synthetase	1	1.89	
22	MPV17	MpV17 mitochondrial inner membrane protein	1	1.68	
23	ATOX1	ATX1 antioxidant protein 1 homolog (yeast)	1	1.86	
24	CCL5	Chemokine (C–C motif) ligand 5	1	0.67	
25	MPO	Myeloperoxidase	1	1.58	

## Discussion

Recently, we reported the therapeutic potential of DBMSCs to treat inflammatory diseases, such as atherosclerosis and cancer [14–16, 19]. Diabetes is another inflammatory diseases [8] where high levels of glucose (an oxidative stress mediator) cause cellular and tissue damage [20–23]. Therefore, for an effective application of DBMSCs in diabetes, it is important for DBMSCs maintain their normal reparative properties when exposed to high levels of glucose. Here, we studied the functional and phenotypic changes of DBMSCs in response to glucose.

First, we examined the effect of different glucose concentrations on the survival of DBMSCs. We report that DBMSCs survive in high levels of 200 mM glucose even when cultured for long periods. DBMSCs also showed increased proliferation potential at high levels of glucose (Fig. 1D–F), via a reversible mechanism (Fig. 1G–I). This contrasts with our previous finding in which MSCs from the chorionic villi of human placentae (pMSCs) show a reduction in their proliferation under the effect of glucose [18]. This may reflect DBMSC adaption to the elevated oxidative stress levels in normal pregnancy, as a result of their vascular microenvironment where they are directly or indirectly exposed to factors in maternal pregnant blood, which contains high level of oxidative stress mediators [24]. On the other hand, pMSCs in the chorionic vascular niche, are exposed to the fetal circulation, which contains relatively reduced levels of oxidative stress throughout normal pregnancy [13, 25].

Glucose also induced DBMSC expression of genes associated with survival, anti-apoptotic [26–37], and proliferation [38–40] as shown in Table 1. These molecules may prevent the damaging effects of glucose on DBMSCs. However, this needs further investigation to confirm the protective roles of these molecules in the survival and proliferation of DBMSCs from glucose. In this study, preconditioning of DBMSCs with glucose enhanced their adhesion (Fig. 2) possibly via ICAM-1 (Fig. 4A). This is consistent with our previous study which showed that DBMSCs preconditioned with H_2_O_2_ showed increased adhesion [14]. Other studies also support our finding, where preconditioning of hematopoietic stem cells with H_2_O_2_ also increased their adhesion *in vitro* and *in vivo* [41]. Adhesion is the first important biological process required for a successful stem cell engraftment [42, 43]. Migration and invasion of MSCs are other important biological processes that occur during MSC engraftment in a disease environment with high level of oxidative stress mediators [42, 43]. We found that DBMSCs preconditioned with glucose improved their migration (Fig. 3D). This effect is similar to the effect of H_2_O_2_ on the migration of DBMSCs [14], MSCs from the chorionic villi [44] and bone marrow [45]. DBMSCs preconditioned with glucose also improved their invasion (Fig. 3E) via a mechanism that may involve the induction of a number of genes known for their migratory [26–29, 31, 36, 46–51] and invasive properties [26–28, 47, 48], Table 1. These results demonstrate that the engraftment properties of DBMSCs can be improved by glucose pretreatment, possibly via these genes. Thus, preconditioning DBMSCs could be valuable component of cell-based therapies that must act in high oxidative stress environments. However, a future mechanistic study is necessary to confirm this further.

In the pancreatic beta islets, the pro-oxidant enzymes (i.e. NOX1-5 and DUOX1-2) increase the production of the reactive oxygen specie (ROS) superoxide, which induces insulin secretion [52–56]. The excessive accumulation of ROS causes beta cell damage, which can be prevented by the antioxidant enzymes (i.e. GPX, CAT and SOD), which act as ROS scavengers, and therefore inhibit insulin secretion [52–56]. In this study, glucose induced and reduced DBMSC expression of genes with pro-oxidant [39, 57, 58] and anti-oxidant properties, respectively [59], Table 2. Thus, indicating that glucose may direct DBMSCs to activate pathways associated with insulin secretion. This postulate is supported by the finding that glucose also induced DBMSC expression of albumin and NOS2, which are associated with insulin secretion [32, 60]. In addition, glucose also reduced DBMSC expression of PXDN, a molecule that triggers diabetes, Table 4 [61].

Generally, a basal level of ROS is required to stimulate basic cellular biological activities (i.e. proliferation, migration, and invasion). ROS is also required for insulin secretion by beta cells. As discussed above, the high level of ROS damages tissue, and consequently this is avoided by the antioxidant enzymes which are produced to scavenger ROS [62]. Glucose simultaneously induced DBMSC expression of both pro-oxidant (Table 4) and anti-oxidant genes [40, 50, 63–66], Table 4. Therefore, DBMSCs may respond to glucose induction of ROS by generating antioxidants to prevent cellular damage and also to regulate insulin secretion probably by inducing the expression of UCP2 (Table 4), which has anti-insulin secretion activity [63].

In diabetes, the oxidative stress mediators generated by the high level of glucose, stimulate the recruitment of immune cells to the site of tissue injury, and this in return will intensify tissue damage [67]. One of the therapeutic strategies, is to reduce the recruitment of immune cells to the injured tissue. In this study, glucose reduced DBMSCs expression of thioredoxin (Table 4), an oxidative stress molecule that increases the recruitment of immune cells [67]. Glucose also increased the anti-inflammatory properties of DBMSCs by increasing their expression of anti-inflammatory genes [26, 31, 34, 35, 63, 64, 68–74] (Table 3), and also by reducing their expression of pro-inflammatory genes including MGST3 and COX2 [75–77]. This finding is important, because these anti-inflammatory molecules reduce the recruitment of immune cells [70]. These results indicate that DBMSCs may function as an anti-chemoattractant agent to reduce the recruitment of immune cells to the injured tissues in inflammatory diseases. The property of DBMSCs to ameliorate inflammation is further confirmed by their enhanced expression of B7-H4 in response to glucose (Fig. 4). B7-H4 is a protein that inhibits T-cell proliferation [78], suggesting that DBMSCs may inhibit T cell proliferation, and thus reduce inflammation.

High levels of glucose also causes immune dysfunction that results in a reduction in the antimicrobial activity of the immune cells in diabetic patients, and therefore these patients are at a higher risk to bacterial infection [79]. In this study, glucose induced DBMSC expression of antibacterial genes, [71, 73], Table 3. Collectively, these results suggest that DBMSCs may have the potential to treat inflammatory diseases, such as diabetes, by ameliorating inflammation and also by preventing infection associated with hyperglycaemia.

**Fig. 5:**
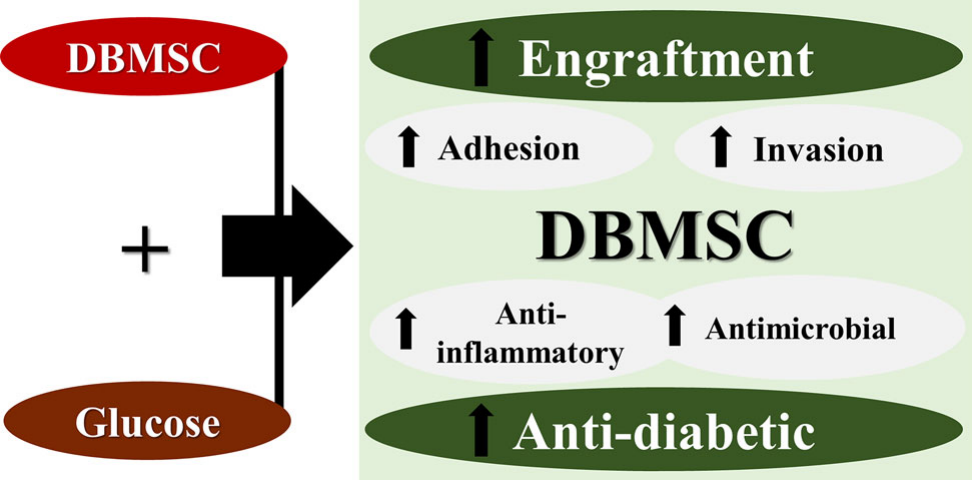
Illustration showing the treatment of DBMSCs with glucose increases their functional (adhesion, invasion and engraftment) and phenotypic (anti-inflammatory, anti-microbial and anti-diabetic) properties

This is the first study to show the beneficial effects of glucose on DBMSC functions. Preconditioning of DBMSCs with glucose may increase their therapeutic potential by enhancing their engraftment efficiency (Fig. 5). In addition, the induction of ROS in DBMSCs by glucose may program these cells into insulin producing cells with ability to counteract inflammation and infection associated with diabetes (Fig. 5). However, future *in vitro* and *in vivo* studies are essential to investigate the findings of this study further.

## Electronic supplementary material

Below is the link to the electronic supplementary material.

Supplementary material 1 (DOCX 188 kb)

Supplementary material 2 (DOCX 12 kb)

## References

[1] Abomaray FM, Al Jumah MA, Alsaad KO, Jawdat D, Al Khaldi A, AlAskar AS (2016). Phenotypic and functional characterization of mesenchymal stem/multipotent stromal cells from decidua basalis of human term placenta. Stem Cells Int.

[2] Abumaree MH, Abomaray FM, Alshehri NA, Almutairi A, Al-Askar AS, Kalionis B (2016). Phenotypic and functional characterization of mesenchymal stem/multipotent stromal cells from decidua parietalis of human term placenta. Reprod Sci.

[3] Abumaree MH, Al Jumah MA, Kalionis B, Jawdat D, Al Khaldi A, Al Talabani AA (2013). Phenotypic and functional characterization of mesenchymal stem cells from chorionic villi of human term placenta. Stem Cell Rev Rep.

[4] Abomaray FM, Al Jumah MA, Kalionis B, AlAskar AS, Al Harthy S, Jawdat D (2015). Human chorionic villous mesenchymal stem cells modify the functions of human dendritic cells, and induce an anti-inflammatory phenotype in CD1+ dendritic cells. Stem Cell Rev Rep.

[5] Abumaree MH, Abomaray FM, Alshabibi MA, AlAskar AS, Kalionis B (2017). Immunomodulatory properties of human placental mesenchymal stem/stromal cells. Placenta.

[6] Abumaree MH, Al Jumah MA, Kalionis B, Jawdat D, Al Khaldi A, Abomaray FM (2013). Human placental mesenchymal stem cells (pMSCs) play a role as immune suppressive cells by shifting macrophage differentiation from inflammatory M1 to anti-inflammatory M2 macrophages. Stem Cell Rev Rep.

[7] Al Jumah MA, Abumaree MH (2012). The immunomodulatory and neuroprotective effects of mesenchymal stem cells (MSCs) in experimental autoimmune encephalomyelitis (EAE): a model of multiple sclerosis (MS). Int J Mol Sci.

[8] Zang L, Hao H, Liu J, Li Y, Han W, Mu Y (2017). Mesenchymal stem cell therapy in type 2 diabetes mellitus. Diabetol Metab Syndr.

[9] Li F, Guo X, Chen SY (2017). Function and therapeutic potential of mesenchymal stem cells in atherosclerosis. Front Cardiovasc Med.

[10] de Mendonca L, Felix NS, Blanco NG, Da Silva JS, Ferreira TP, Abreu SC (2017). Mesenchymal stromal cell therapy reduces lung inflammation and vascular remodeling and improves hemodynamics in experimental pulmonary arterial hypertension. Stem Cell Res Ther.

[11] Auletta JJ, Cooke KR, Solchaga LA, Deans RJ, van’t Hof W (2010). Regenerative stromal cell therapy in allogeneic hematopoietic stem cell transplantation: current impact and future directions. Biol Blood Marrow Transplant.

[12] Kanasaki K, Kalluri R (2009). The biology of preeclampsia. Kidney Int.

[13] Kusuma GD, Abumaree MH, Pertile MD, Perkins AV, Brennecke SP, Kalionis B (2016). Mesenchymal stem/stromal cells derived from a reproductive tissue niche under oxidative stress have high aldehyde dehydrogenase activity. Stem Cell Rev Rep.

[14] Khatlani T, Algudiri D, Alenzi R, Al Subayyil AM, Abomaray FM, Bahattab E (2018). Preconditioning by hydrogen peroxide enhances multiple properties of human decidua basalis mesenchymal stem/multipotent stromal cells. Stem Cells Int.

[15] Alshabibi MA, Khatlani T, Abomaray FM, AlAskar AS, Kalionis B, Messaoudi SA (2018). Human decidua basalis mesenchymal stem/stromal cells protect endothelial cell functions from oxidative stress induced by hydrogen peroxide and monocytes. Stem Cell Res Ther.

[16] Alshabibi MA, Al Huqail AJ, Khatlani T, Abomaray FM, Alaskar AS, Alawad AO (2017). Mesenchymal stem/multipotent stromal cells from human decidua basalis reduce endothelial cell activation. Stem Cells Dev.

[17] Abumaree MH, Hakami M, Abomaray FM, Alshabibi MA, Kalionis B, Al Jumah MA (2017). Human chorionic villous mesenchymal stem/stromal cells modify the effects of oxidative stress on endothelial cell functions. Placenta.

[18] Basmaeil YS, Al Subayyil AM, Khatlani T, Bahattab E, Al-Alwan M, Abomaray FM (2018). Human chorionic villous mesenchymal stem/stromal cells protect endothelial cells from injury induced by high level of glucose. Stem Cell Res Ther.

[19] Abumaree MH, Al Harthy S, Al Subayyil AM, Alshabibi MA, Abomaray FM, Khatlani T (2019). Decidua basalis mesenchymal stem cells favor inflammatory M1 macrophage differentiation in vitro. Cells.

[20] Paneni F, Beckman JA, Creager MA, Cosentino F (2013). Diabetes and vascular disease: pathophysiology, clinical consequences, and medical therapy: part I. Eur Heart J.

[21] Giacco F, Brownlee M (2010). Oxidative stress and diabetic complications. Circ Res.

[22] Tabit CE, Chung WB, Hamburg NM, Vita JA (2010). Endothelial dysfunction in diabetes mellitus: molecular mechanisms and clinical implications. Rev Endocr Metab Disord.

[23] Carr ME (2001). Diabetes mellitus: a hypercoagulable state. J Diabetes Complications.

[24] Raijmakers MT, Roes EM, Poston L, Steegers EA, Peters WH (2008). The transient increase of oxidative stress during normal pregnancy is higher and persists after delivery in women with pre-eclampsia. Eur J Obstet Gynecol Reprod Biol.

[25] Braekke K, Harsem NK, Staff AC (2006). Oxidative stress and antioxidant status in fetal circulation in preeclampsia. Pediatr Res.

[26] Kost OA, Beznos OV, Davydova NG, Manickam DS, Nikolskaya II, Guller AE (2015). Superoxide dismutase 1 nanozyme for treatment of eye inflammation. Oxid Med Cell Longev.

[27] Niu W, Zhang M, Chen H, Wang C, Shi N, Jing X (2016). Peroxiredoxin 1 promotes invasion and migration by regulating epithelial-to-mesenchymal transition during oral carcinogenesis. Oncotarget.

[28] Taniuchi K, Furihata M, Hanazaki K, Iwasaki S, Tanaka K, Shimizu T (2015). Peroxiredoxin 1 promotes pancreatic cancer cell invasion by modulating p38 MAPK activity. Pancreas.

[29] Park KR, Yun HM, Yeo IJ, Cho S, Hong JT, Jeong YS (2018). Peroxiredoxin 6 inhibits osteogenic differentiation and bone formation through human dental pulp stem cells and induces delayed bone development. Antioxid Redox Signal.

[30] Zhou Y, Cao X, Yang Y, Wang J, Yang W, Ben P (2018). Glutathione S-transferase Pi prevents sepsis-related high mobility group box-1 protein translocation and release. Front Immunol.

[31] Wenzel P, Rossmann H, Müller C, Kossmann S, Oelze M, Schulz A (2015). Heme oxygenase-1 suppresses a pro-inflammatory phenotype in monocytes and determines endothelial function and arterial hypertension in mice and humans. Eur Heart J.

[32] Meivar-Levy I, Sapir T, Berneman D, Weissbach T, Polak-Charcon S, Ravassard P (2011). Human liver cells expressing albumin and mesenchymal characteristics give rise to insulin-producing cells. J Transplant.

[33] Oliver PL, Finelli MJ, Edwards B, Bitoun E, Butts DL, Becker EB (2011). Oxr1 is essential for protection against oxidative stress-induced neurodegeneration. PLoS Genet.

[34] Roth W, Kumar V, Beer HD, Richter M, Wohlenberg C, Reuter U (2012). Keratin 1 maintains skin integrity and participates in an inflammatory network in skin through interleukin-18. J Cell Sci.

[35] Zhu H, Li Y (2012). NAD(P)H: quinone oxidoreductase 1 and its potential protective role in cardiovascular diseases and related conditions. Cardiovasc Toxicol.

[36] Preisinger C, Short B, De Corte V, Bruyneel E, Haas A, Kopajtich R (2004). YSK1 is activated by the Golgi matrix protein GM130 and plays a role in cell migration through its substrate 14-3-3zeta. J Cell Biol.

[37] Hendgen-Cotta UB, Esfeld S, Coman C, Ahrends R, Klein-Hitpass L, Flögel U (2017). A novel physiological role for cardiac myoglobin in lipid metabolism. Sci Rep.

[38] Davis DB, Lavine JA, Suhonen JI, Krautkramer KA, Rabaglia ME, Sperger JM (2010). FoxM1 is up-regulated by obesity and stimulates beta-cell proliferation. Mol Endocrinol.

[39] Cho KJ, Seo JM, Kim JH (2011). Bioactive lipoxygenase metabolites stimulation of NADPH oxidases and reactive oxygen species. Mol Cells.

[40] Shen Y, Nar R, Fan AX, Aryan M, Hossain MA, Gurumurthy A (2018). Functional interrelationship between TFII-I and E2F transcription factors at specific cell cycle gene loci. J Cell Biochem.

[41] Kavanagh DP, Yemm AI, Alexander JS, Frampton J, Kalia N (2013). Enhancing the adhesion of hematopoietic precursor cell integrins with hydrogen peroxide increases recruitment within murine gut. Cell Transplant.

[42] Veevers-Lowe J, Ball SG, Shuttleworth A, Kielty CM (2011). Mesenchymal stem cell migration is regulated by fibronectin through alpha5beta1-integrin-mediated activation of PDGFR-beta and potentiation of growth factor signals. J Cell Sci.

[43] Frijns CJ, Kappelle LJ (2002). Inflammatory cell adhesion molecules in ischemic cerebrovascular disease. Stroke.

[44] Oh JY, Choi GE, Lee HJ, Jung YH, Ko SH, Chae CW (2018). High glucose-induced reactive oxygen species stimulates human mesenchymal stem cell migration through snail and EZH2-dependent E-cadherin repression. Cell Physiol Biochem.

[45] Li S, Deng Y, Feng J, Ye W (2009). Oxidative preconditioning promotes bone marrow mesenchymal stem cells migration and prevents apoptosis. Cell Biol Int.

[46] Kwon J, Wang A, Burke DJ, Boudreau HE, Lekstrom KJ, Korzeniowska A (2016). Peroxiredoxin 6 (Prdx6) supports NADPH oxidase1 (Nox1)-based superoxide generation and cell migration. Free Radic Biol Med.

[47] Zhong C, Zhuang M, Wang X, Li J, Chen Z, Huang Y (2018). 12-Lipoxygenase promotes invasion and metastasis of human gastric cancer cells via epithelial-mesenchymal transition. Oncol Lett.

[48] Klampfl T, Bogner E, Bednar W, Mager L, Massudom D, Kalny I (2012). Up-regulation of 12(S)-lipoxygenase induces a migratory phenotype in colorectal cancer cells. Exp Cell Res.

[49] Maes H, Van Eygen S, Krysko DV, Vandenabeele P, Nys K, Rillaerts K (2014). BNIP3 supports melanoma cell migration and vasculogenic mimicry by orchestrating the actin cytoskeleton. Cell Death Dis.

[50] Tol MJ, Ottenhoff R, van Eijk M, Zelcer N, Aten J, Houten SM (2016). A PPARgamma-Bnip3 axis couples adipose mitochondrial fusion-fission balance to systemic insulin sensitivity. Diabetes.

[51] Nerstedt A, Cansby E, Andersson CX, Laakso M, Stančáková A, Blüher M (2012). Serine/threonine protein kinase 25 (STK25): a novel negative regulator of lipid and glucose metabolism in rodent and human skeletal muscle. Diabetologia.

[52] Simões D, Riva P, Peliciari-Garcia RA, Cruzat VF, Graciano MF, Munhoz AC (2016). Melatonin modifies basal and stimulated insulin secretion via NADPH oxidase. J Endocrinol.

[53] Morgan D, Rebelato E, Abdulkader F, Graciano MF, Oliveira-Emilio HR, Hirata AE (2009). Association of NAD(P)H oxidase with glucose-induced insulin secretion by pancreatic beta-cells. Endocrinology.

[54] Pi J, Bai Y, Zhang Q, Wong V, Floering LM, Daniel K (2007). Reactive oxygen species as a signal in glucose-stimulated insulin secretion. Diabetes.

[55] Bedard K, Krause KH (2007). The NOX family of ROS-generating NADPH oxidases: physiology and pathophysiology. Physiol Rev.

[56] Newsholme P, Morgan D, Rebelato E, Oliveira-Emilio HC, Procopio J, Curi R (2009). Insights into the critical role of NADPH oxidase(s) in the normal and dysregulated pancreatic beta cell. Diabetologia.

[57] O’Neill S, Brault J, Stasia MJ, Knaus UG (2015). Genetic disorders coupled to ROS deficiency. Redox Biol.

[58] Kundu TK, Hille R, Velayutham M, Zweier JL (2007). Characterization of superoxide production from aldehyde oxidase: an important source of oxidants in biological tissues. Arch Biochem Biophys.

[59] Pellegrinelli V, Rouault C, Rodriguez-Cuenca S, Albert V, Edom-Vovard F, Vidal-Puig A (2015). Human adipocytes induce inflammation and atrophy in muscle cells during obesity. Diabetes.

[60] Henningsson R, Salehi A, Lundquist I (2002). Role of nitric oxide synthase isoforms in glucose-stimulated insulin release. Am J Physiol Cell Physiol.

[61] Brown KL, Darris C, Rose KL, Sanchez OA, Madu H, Avance J (2015). Hypohalous acids contribute to renal extracellular matrix damage in experimental diabetes. Diabetes.

[62] Jiao Y, Wang Y, Guo S, Wang G (2017). Glutathione peroxidases as oncotargets. Oncotarget.

[63] Chan CB, Kashemsant N (2006). Regulation of insulin secretion by uncoupling protein. Biochem Soc Trans.

[64] Barrett CW, Short SP, Williams CS (2017). Selenoproteins and oxidative stress-induced inflammatory tumorigenesis in the gut. Cell Mol Life Sci.

[65] Parsons JL, Khoronenkova SV, Dianova II, Ternette N, Kessler BM, Datta PK (2012). Phosphorylation of PNKP by ATM prevents its proteasomal degradation and enhances resistance to oxidative stress. Nucleic Acids Res.

[66] Bafort F, Parisi O, Perraudin JP, Jijakli MH (2014). Mode of action of lactoperoxidase as related to its antimicrobial activity: a review. Enzyme Res.

[67] Bertini R, Howard OM, Dong HF, Oppenheim JJ, Bizzarri C, Sergi R (1999). Thioredoxin, a redox enzyme released in infection and inflammation, is a unique chemoattractant for neutrophils, monocytes, and T cells. J Exp Med.

[68] Inoue K, Takano H, Shimada A, Satoh M (2009). Metallothionein as an anti-inflammatory mediator. Mediators Inflamm.

[69] Couto N, Wood J, Barber J (2016). The role of glutathione reductase and related enzymes on cellular redox homoeostasis network. Free Radic Biol Med.

[70] Laurila JP, Laatikainen LE, Castellone MD, Laukkanen MO (2009). SOD3 reduces inflammatory cell migration by regulating adhesion molecule and cytokine expression. PLoS One.

[71] Sorensen GL (2018). Surfactant protein D in respiratory and non-respiratory diseases. Front Med (Lausanne).

[72] Zhang X, Hyer JM, Yu H, D’Silva NJ, Kirkwood KL (2014). DUSP1 phosphatase regulates the proinflammatory milieu in head and neck squamous cell carcinoma. Cancer Res.

[73] Stienstra R, Dijk W, van Beek L, Jansen H, Heemskerk M, Houtkooper RH (2014). Mannose-binding lectin is required for the effective clearance of apoptotic cells by adipose tissue macrophages during obesity. Diabetes.

[74] Buechler N, Wang X, Yoza BK, McCall CE, Vachharajani V (2017). Sirtuin 2 regulates microvascular inflammation during sepsis. J Immunol Res.

[75] Thameem F, Yang X, Permana PA, Wolford JK, Bogardus C, Prochazka M (2003). Evaluation of the microsomal glutathione S-transferase 3 (MGST3) locus on 1q23 as a type 2 diabetes susceptibility gene in Pima Indians. Hum Genet.

[76] Ford-Hutchinson AW (1990). Leukotriene B4 in inflammation. Crit Rev Immunol.

[77] Cha YI, DuBois RN (2007). NSAIDs and cancer prevention: targets downstream of COX-2. Annu Rev Med.

[78] Ou D, Wang X, Metzger DL, Ao Z, Pozzilli P, James RF (2006). Suppression of human T-cell responses to beta-cells by activation of B7-H4 pathway. Cell Transplant.

[79] Casqueiro J, Casqueiro J, Alves C (2012). Infections in patients with diabetes mellitus: a review of pathogenesis. Indian J Endocrinol Metab.

