# Correction to: Preconditioning of Human Decidua Basalis Mesenchymal Stem/Stromal Cells with Glucose Increased Their Engraftment and Anti-diabetic Properties

**DOI:** 10.1007/s13770-020-00251-x

**Published:** 2020-05-03

**Authors:** Yasser Basmaeil, Manar Al Rashid, Tanvir Khatlani, Manal AlShabibi, Eman Bahattab, Meshan L. Abdullah, Fawaz Abomaray, Bill Kalionis, Safia Massoudi, Mohamed Abumaree

**Affiliations:** 1 Stem Cells and Regenerative Medicine Department, King Abdullah International Medical Research Center, King Abdulaziz Medical City, Ministry of National Guard Health Affairs, Mail Code 1515, P.O. Box 22490, Riyadh, 11426 Kingdom of Saudi Arabia; 2 National Center for Stem Cell Technology, Life Sciences and Environment Research Institute, King Abdulaziz City for Science and Technology, P.O Box 6086, Riyadh, 11442 Kingdom of Saudi Arabia; 3 Experimental Medicine, King Abdullah International Medical Research Center MNG-HA, Ali Al Arini, Ar Rimayah, Riyadh, 11481 Kingdom of Saudi Arabia; 4 Division of Obstetrics and Gynecology, Department of Clinical Science, Intervention and Technology, Karolinska Institutet, 14186 Stockholm, Sweden; 5 Department of Maternal–Fetal Medicine, Pregnancy Research Centre and University of Melbourne, Parkville, VIC 3010 Australia; 6 Department of Obstetrics and Gynaecology, Royal Women’s Hospital, 20 Flemington Rd, Parkville, VIC 3052 Australia; 7 Department of Forensic Biology, College of Forensic Sciences, Naif Arab University for Security Sciences, Khurais Rd, Ar Rimayah, Riyadh, 14812 Kingdom of Saudi Arabia; 8 College of Science and Health Professions, King Saud Bin Abdulaziz University for Health Sciences, King Abdulaziz Medical City, Ministry of National Guard Health Affairs, Mail Code 3124, P.O. Box 3660, Riyadh, 11481 Kingdom of Saudi Arabia

## Correction to: Tissue Eng Regen Med 10.1007/s13770-020-00239-7

The author would like to correct the names for the below co-authors in the online published article.

The correct author names are given below:

Fawaz Abomaray

Mohamed Abumaree

