# Supplementary material for: Preconditioning of Human Decidua Basalis Mesenchymal Stem/Stromal Cells with Glucose Increased Their Engraftment and Anti-diabetic Properties
Source: Tissue Eng Regen Med. 2020 Feb 19;17(2):209–22. doi: 10.1007/s13770-020-00239-7 (PMC7105536; doi:10.1007/s13770-020-00239-7)
Supplement: Supplementary file 1 — Supplementary material 1 (DOCX 188 kb) [file 13770_2020_239_MOESM1_ESM.docx]

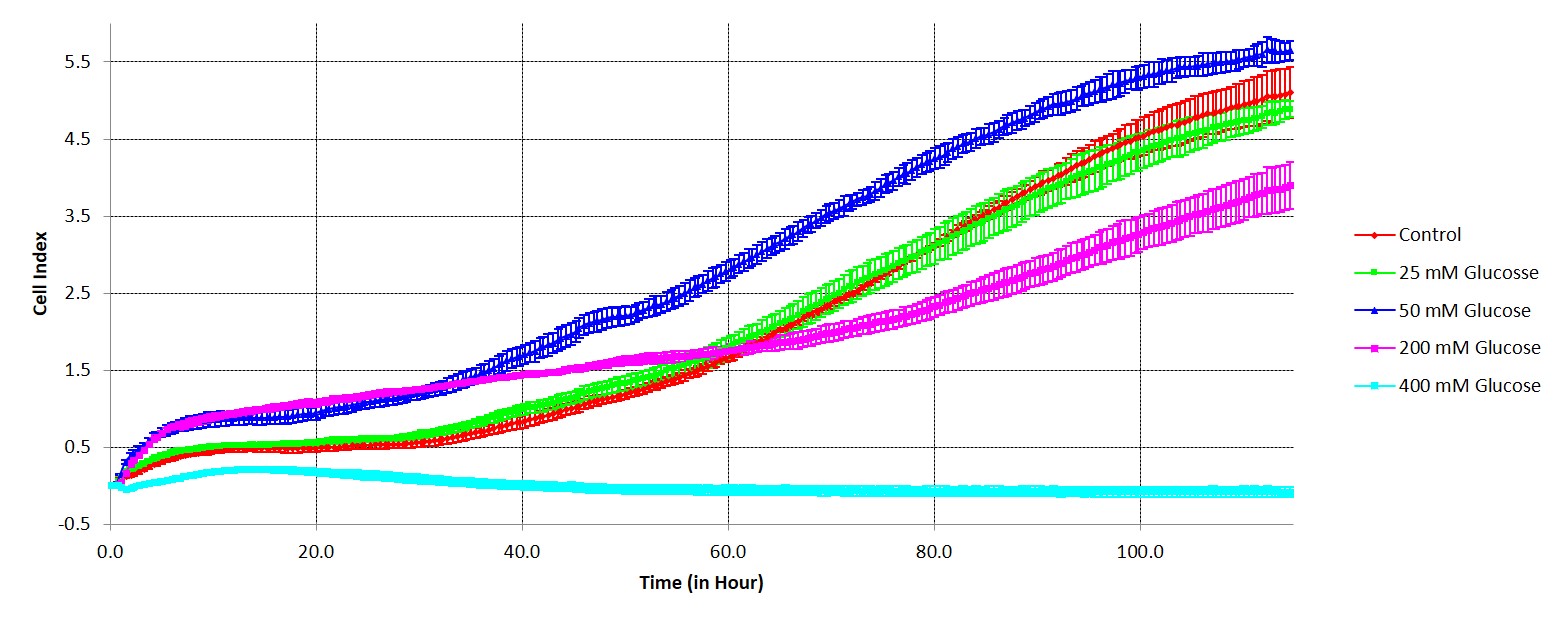


**Supplementary figure 1.** Proliferation of DBMSC in high glucose condition for 120 hours using xCelligence Real time analyzer
