# Supplementary material for: Preconditioning of Human Decidua Basalis Mesenchymal Stem/Stromal Cells with Glucose Increased Their Engraftment and Anti-diabetic Properties
Source: Tissue Eng Regen Med. 2020 Feb 19;17(2):209–22. doi: 10.1007/s13770-020-00239-7 (PMC7105536; doi:10.1007/s13770-020-00239-7)
Supplement: Supplementary file 2 — Supplementary material 2 (DOCX 12 kb) [file 13770_2020_239_MOESM2_ESM.docx]

**Supplementary Table 1:** **DBMSC treatment groups used in the proliferation and adhesion experiments.**

| Groups | Description |
| --- | --- |
| 1 | DBMSC cultured alone |
| 2 | DBMSC cultured with different concentrations (25-400 mM) of glucose |
| 3 | 200 (Pre) [DBMSCs precultured with 200mM glucose for 72 h]. DBMSCs were then harvested and used. |

**Supplementary Table 2 DBMSC treatment groups used in the migration experiments.**

| Groups | Description |
| --- | --- |
| 1 | DB [DBMSCs cultured alone]. |
| 2 | DB (T 200) [DBMSCs cultured in the upper chamber while 200mM/ ml glucose was added to the lower chamber]. |
| 3 | Pre-DB [DBMSCs precultured with 200mM/ ml glucose for 72 h] cultured alone in the upper chamber while 200mM/ ml glucose was added to the lower chamber. |

**Supplementary Table 3 DBMSC treatment groups used in the invasion experiments.**

| Groups | Description |
| --- | --- |
| 1 | DBMSCs were added to the monolayer of endothelial cells. |
| 2 | 200 (in) [DBMSCs were added in the presence of 200 mM of glucose to the monolayer of endothelial cells]. |
| 3 | 200 (pre) [DBMSCs Precultured with 200mM glucose for 72 h]. DBMSCs were then harvested and added to the monolayer of endothelial cells. |
